# Frailty and Mortality Risk Among Dogs with Extreme Longevity: Development and Predictive Validity of a Clinical Frailty Index in the Exceptional Aging in Rottweilers Study

**DOI:** 10.3390/ani14243651

**Published:** 2024-12-18

**Authors:** David J. Waters, Aimee H. Maras, Rong Fu, Andres E. Carrillo, Emily C. Chiang, Cheri L. Suckow

**Affiliations:** 1 Center for Exceptional Longevity Studies, Gerald P. Murphy Cancer Foundation, West Lafayette, IN 47906, USA; ahmaras@aol.com (A.H.M.); emilychiang1225@gmail.com (E.C.C.); cheri@gpmcf.org (C.L.S.); 2 Center on Aging and the Life Course and the Department of Veterinary Clinical Sciences, Purdue University, West Lafayette, IN 47907, USA; 3 Department of Sociology, Siena College, Loudonville, NY 12211, USA; rfu@siena.edu; 4 Department of Exercise Science, College of Health Sciences, Chatham University, Pittsburgh, PA 15232, USA; acarrillo@chatham.edu

**Keywords:** dog aging, longevity, gerontology, healthspan, deficit accumulation, frailty limit, comparative biology, centenarians, companion dog

## Abstract

Frailty refers to a state of increased vulnerability to mortality and other adverse outcomes as a consequence of age-related decline in physiologic reserve and function. Comparative biomedical scientists are relied upon to innovate approaches to enhance understanding of the similarities and differences between humans and other animal species that can impact healthy aging. The research aim of this study was to develop a clinical frailty index (FI) in the Exceptional Aging in Rottweilers Study (EARS) and test its ability to predict all-cause mortality in elderly dogs. EARS is an ongoing lifetime cohort study of pet dogs with extreme longevity living in North America. Living 30% longer than the breed average, these dogs represent the canine counterpart to human centenarians. A 34-item FI (EARS-FI) was constructed to assess deficit accumulation using clinical data collected by telephone interviews with owners of 93 dogs with extreme longevity. Health deficits across multiple domains, including cognitive and sensory, cardiovascular and endocrine, and mobility, were included. The association between EARS-FI and subsequent mortality was tested in Kaplan-Meier survival analysis and in age-adjusted Cox proportional hazard models. Median (interquartile range) EARS-FI was 0.43 (0.38–0.50), and the estimated frailty limit was 0.68, consistent with data reported in humans with extreme longevity. Frailty index increased with increasing chronological age (*p* < 0.001). Deficit accumulation was significantly associated with increased mortality risk. Age-adjusted hazard ratio for mortality per 0.01 unit increase in FI was 1.05 (95%CI, 1.02–1.08; *p* = 0.001). This work provides the first demonstration of a strong association between frailty and mortality risk in pet dogs with extreme longevity. Notably, EARS-FI showed key features observed in the evaluation of frailty in aging human populations: heterogeneity, increase with chronological age, and estimated limit of <0.7. Validated here as a predictor of mortality in aged pet dogs, EARS-FI offers a useful tool for further comparative analyses of the linkages between deficit accumulation, mortality, and other adverse health outcomes.

## Simple Summary

Within the elderly dog population, not all dogs of the same breed at the same age have the same vulnerability to the future development of adverse health outcomes. Understanding this biological heterogeneity is one key to advancing the health of geriatric pets. The quantitation of an individual’s health deficits using a clinical tool called frailty index (FI) has been applied to aging human populations. Two previous studies have reported FI in dogs; neither focused on geriatric dogs. Our aim was to capitalize on The Exceptional Aging in Rottweilers Study (EARS), the first study of pet dogs living in North America that have reached extreme longevity, by developing an FI in geriatric dogs and then testing this tool as a predictor of mortality. We constructed a clinical frailty index, EARS-FI, using information attained through telephone interviews with owners of 93 dogs. Using EARS-FI, we showed these dogs have a broad range of FI values, an increase in frailty with increasing age, and an upper FI limit. Notably, we provide the first demonstration of increased mortality risk associated with increasing frailty in pet dogs with extreme longevity. The work has important implications for both pet owners and One Health investigators. EARS-FI should provide a useful tool for further in-depth analyses of linkages between health deficit accumulation and adverse clinical outcomes. This research benefits geriatric pets by contributing to a better understanding of frailty in aging dogs and sets the stage for identifying factors that undermine optimal trajectories of lifetime physical health.

## 1. Introduction

Mortality risk in biological systems increases with age, yet the magnitude of this risk is heterogeneous across aging populations [1]. In people, ‘frail’ is the descriptor given to those individuals with a higher vulnerability to mortality or other adverse outcomes, such as falling, prolonged hospitalization, or incident dementia [2,3]. The two most common frailty assessment tools are frailty phenotype (based on five phenotypic criteria) and frailty index (based on deficit accumulation) [4].

Frailty index (FI) operationalizes frailty as deficit accumulation, i.e., the proportion of health deficits present in each individual, thereby offering vital insights into the aging process and its consequences in terms of predicting healthy life expectancy and mortality risk [1,4,5,6,7]. Frailty index based upon collection of data through clinical interviews and evaluations (referred to as Clinical FI) and FI based on results of commonly run laboratory tests (referred to as Lab-FI) both predict mortality risk in humans [8], suggesting that determining the accumulation of health-relevant deficits—deficits ranging from biochemical to outwardly detectable—provides a useful tool for promoting a more nuanced understanding of biological aging.

Over the last decade, the need to develop measures of frailty in other animal species has been emphasized [9,10,11]. Numerous reports from laboratory mice have shown FI can be used to (1) usefully quantify frailty in aged individuals and show that FI increases exponentially with age [12]; (2) demonstrate the relationship between increasing frailty and shortened survival [1]; and (3) assess the effect of healthspan-promoting interventions, such as resveratrol or caloric restriction, on the reduction of frailty scores [13]. Investigative approaches leading to the development of frailty models have been reviewed [14,15].

There is growing interest in harnessing the domesticated dog population as an underutilized opportunity to better understand the genetic and non-genetic determinants of healthy aging [16,17,18,19,20,21,22]. Banzato and colleagues [23] developed a clinical FI and tested its association with mortality in 401 dogs of more than 30 breeds. In their hands, FI increased with chronological age, and deficit accumulation was strongly related to 6-month mortality. The estimated FI limit was 0.59, and no sex differences were detected. Other reports of canine frailty have focused on frailty phenotype [24,25,26].

The Exceptional Aging in Rottweilers Study (EARS) seeks to better understand the biology of cellular aging and disease resistance by discovering genetic and environmental correlates of morbidity compression and longevity through the detailed study of the oldest living Rottweilers in North America. Living 30% longer than the breed average, these dogs represent the canine counterpart to human centenarians. The ambidirectional cohort study design of EARS—consisting of both retrospective and prospective acquisition of extensive data from dogs reaching extreme longevity—includes a subset of 93 dogs whose owners underwent standardized telephone interviews to construct a clinical FI. Although the study of people who reach extreme longevity is recognized as a valuable method to probe the biology of aging [27], FI in human centenarians has been characterized in only a limited number of reports [5,28,29].

What is the strength of association between FI and mortality risk among individuals who reach extreme longevity? How much heterogeneity in frailty is retained among pet dogs with extreme longevity? To pursue these research questions, we utilized our cohort of dogs with extreme longevity, capitalizing on the first systematic study of the oldest living Rottweiler dogs in North America.

Here, we report the development of a 34-item clinical FI in 93 dogs with extreme longevity in the EARS study. We show that EARS-FI, an indicator of deficit accumulation, is significantly associated with increased mortality risk in dogs with extreme longevity. Our results prepare the ground for using EARS-FI to identify factors that can buffer the adverse impact of frailty on mortality and to use EARS-FI as an outcome measure to assess determinants of overall robustness, thereby strengthening the ability to conduct scientifically rigorous studies of healthy longevity in pet dogs.

## 2. Materials and Methods

### 2.1. Study Population and Design

The Exceptional Aging in Rottweilers Study (EARS) is an ongoing, ambidirectional cohort study of client-owned Rottweiler dogs with extreme longevity living in North America [30]. Dogs enrolled in the study meet the following inclusion criteria: (1) validation of purebred status through the American Kennel Club (AKC) registry database; (2) age > 13 years, which represents living more than 30% longer than the average lifespan of the Rottweiler breed [31,32]; and (3) owner willingness to provide information by questionnaire, medical records, and telephone interviews so that lifetime medical histories can be constructed. In addition to this retrospective collection of information, there is a prospective phase of data acquisition, which includes the collection and biobanking of clinical samples and DNA, and the collection of tissue samples at necropsy. Since 2003, more than 400 Rottweilers with extreme longevity have been enrolled. Eligible for the current analysis are the 93 dogs whose owners underwent standardized telephone interviews to construct a clinical FI. More detailed information on study enrollment has been published elsewhere [30]. All procedures were carried out in accordance with the Institutional Animal Care and Use Committees of Purdue University and The Gerald P. Murphy Cancer Foundation.

### 2.2. Construction of EARS-FI, a Clinical Frailty Index (FI) in the Exceptional Aging in Rottweilers Study (EARS)

EARS-FI was constructed using owner responses from a standardized telephone interview. The interview collected responses related to 34 clinical variables to determine deficit accumulation in each dog. All interviews were conducted by a single evaluator (AHM), a veterinarian experienced in canine medicine and age-related declines in the physical function of pet dogs. All dogs were alive at the time of interview, and the age of each dog at frailty scoring was recorded. With the goal of constructing a clinical FI that would be age-sensitive and reflect multiple organ systems, the 34 clinical variables selected satisfied the following criteria: (1) a health deficit that was related to an adverse health status; (2) a deficit whose prevalence generally increases with chronological age; and collectively (3) an array of deficits that reflected perturbation of a range of organ systems; and (4) 30 or more health variables considered in the computation [6].

The 34 deficits used to construct EARS-FI are shown in Table 1. Variables evaluated the following domains: appetite, strength and stamina, sensory (eyesight, hearing), infection, urine and fecal continence, sleep, mobility and balance, level of physical activity, mentation, cognition, pain, body condition, hair coat, current disease conditions (endocrine, cardiac, malignant neoplasia), and overall health trajectory. Most variables were assigned a deficit score of 0 (deficit absent) or 1 (deficit present); for a few variables, scoring of 0.5 was possible if the deficit was mild (equivocal) or controlled with medication. 

For some variables, the scoring of ‘deficit present’ was based upon comparison with the dog’s function as a young adult, i.e., 3 to 5 years old. Frailty index (FI) was calculated as follows: FI equals the sum of health deficits divided by the number of health deficits evaluated. For example, FI calculated for a dog with a total of 16 health deficits would be 16/34 or 0.47.

### 2.3. Evaluation of the Association Between EARS-FI and All-Cause Mortality

Risk of mortality was estimated using interval to death after frailty scoring. Interval to death was calculated using the date of frailty scoring by telephone interview with dog owners and the date of each dog’s death/euthanasia obtained through owner questionnaire or review of medical records by research personnel.

### 2.4. Statistical Analysis

Data analyses were performed using STATA Version 17 (Stata Corp., College Station, TX, USA). Statistical significance was defined as *p* < 0.05, and all tests were two-sided. Descriptive characteristics for groups were expressed as medians (interquartile range (IQR), range) or proportions (%) and compared using Chi-square and independent-sample median tests. A frequency distribution plot was constructed to visually display the heterogeneity of FI values in the study cohort. Estimated frailty limit was defined as 99% of FI values [33]. To characterize the relationship between FI and chronological age, log FI was plotted versus age at frailty scoring, as described by Rockwood and Mitnitski [34]. The slope of this plot enabled an estimation of the extent to which log-transformed deficit accumulation increased per year.

To determine the association between EARS-FI and risk of mortality, Kaplan-Meier survival analysis was used to compare subgroups stratified by tertiles of FI: lowest tertile FI (less than 0.38), middle tertile FI (0.38–0.48), and highest tertile of FI (0.49 and above). Differences were tested for significance using the log-rank test. To estimate the risk of mortality associated with increasing deficit accumulation, the Cox proportional hazard model was used to generate hazard ratios (HR). The proportional hazards assumption was not violated in any of the Cox models. The distribution of continuous variables in the data set was evaluated using scatterplots and did not yield any outliers or influential points. HRs and 95% confidence intervals (95% CI) for mortality were age-adjusted and expressed as risk per 0.01 unit increase in FI, according to the method of Rockwood et al. [1]. These estimates of predictive validity were calculated for the overall study sample and for males and females separately. To test the robustness of our main finding that increasing FI was associated with increased mortality risk, a sensitivity analysis was performed by constructing a Cox proportional hazard model for 89 dogs after excluding four dogs with early mortality (i.e., interval from frailty scoring to death less than one month). If removal of dogs with early mortality markedly attenuated the elevated mortality risk associated with increasing FI, this would diminish the future utility of EARS-FI as a predictor of overall mortality.

## 3. Results

### 3.1. General Description of Study Cohort

The study cohort consisted of 93 purebred Rottweiler dogs in the Exceptional Aging in Rottweilers Study (EARS) that underwent standardized calculation of EARS-FI, a clinical frailty index (Table S1). The 93 dogs in this cohort lived in 89 households in 22 U.S states and Canada. Females outnumbered males in the analytic sample: 59 females and 34 males were studied. None of the females and 10 of 34 (29%) males had intact gonads at the time of frailty scoring. Median (IQR) age at frailty scoring was 13.2 (13.1–13.6) years. Median (IQR) age at death was 14.1 (13.7–14.6) years. The most common causes of death were cancer and neurological conditions.

### 3.2. EARS-FI, a Clinical Frailty Index: Median Values, Distribution, Estimated Limit, and Association with Chronological Age

**Figure 1:**
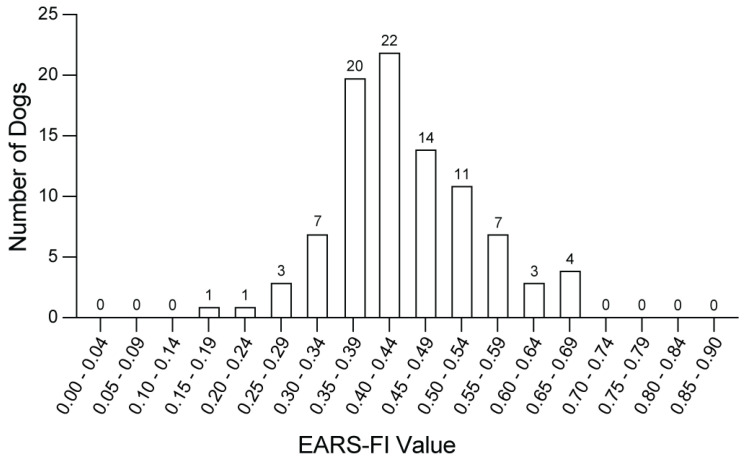
Distribution of values for EARS-FI, a clinical frailty index in the Exceptional Aging in Rottweilers Study (n = 93).

Median and range of FI values are shown in Table 2. Overall, median (IQR) of FI was 0.43 (0.38–0.50). There was no significant difference between median FI of females and males, 0.44 and 0.41, respectively (*p* = 0.89). The distribution of EARS-FI is shown in Figure 1. No dogs had FI < 0.18. Estimated frailty limit, defined as the 99th percentile of FI values, was 0.68.

**Figure 2:**
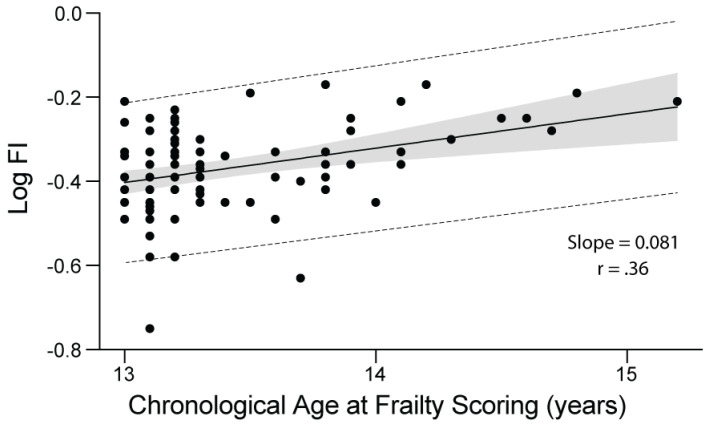
The relationship between deficit accumulation and chronological age in 93 dogs with extreme longevity in the Exceptional Aging in Rottweilers Study. Log FI values are plotted versus age at frailty index (FI) determination. Dots represent FI values for individual dogs. Shaded area represents 95% confidence interval. Dotted lines represent 95% prediction interval.

The relationship between FI and chronological age at frailty scoring is shown in Figure 2. Frailty index increased with increasing chronological age (*p* < 0.001). The overall slope of deficit accumulation versus age was 0.081, indicating that the log-transformed FI score increased, on average, by 8.1% per year.

### 3.3. Deficit Accumulation Is Significantly Associated with Increased Risk of All-Cause Mortality

**Figure 3:**
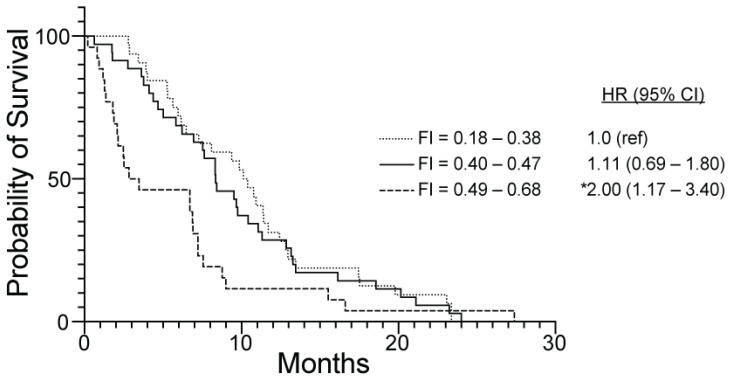
Kaplan–Meier survival curves representing dogs with extreme longevity in the Exceptional Aging in Rottweilers Study (n = 93). Three curves show survival stratified by tertiles of frailty index (FI): low FI (0.18–0.38, n = 32); middle FI (0.40–0.47, n = 35); high FI (0.49–0.68, n = 26). In this survival analysis, time to event is interval from age at frailty scoring to death. Hazard ratio (HR) and 95% confidence interval (95% CI) were generated using Cox proportional hazard modeling using lowest tertile of FI as reference (ref) group (HR = 1.0). * Mortality risk differs significantly from reference group, *p* = 0.01.

Median (range) interval to death after frailty scoring in 93 dogs was 7.6 (0.2–27.4) months. Dogs with higher EARS-FI values had a lower probability of survival. Compared to dogs within the lowest tertile of FI (less than 0.38), dogs within the highest tertile of FI (0.49 and above) had a 2-fold increase in risk of mortality (HR (95% CI) = 2.00 (1.17–3.40); *p* = 0.01) (Figure 3). Overall, Cox proportional hazard modeling showed the age-adjusted HR for mortality per 0.01 unit increase in FI was 1.05 (95% CI, 1.02–1.08; *p* = 0.001) (Table 2). Considering males and females separately, the association between an increase in FI and mortality risk was significant in both sexes (Table 2).

To test further the robustness of the utility of EARS-FI as a predictor of mortality risk, a sensitivity analysis was performed on 89 dogs after exclusion of four dogs with a brief interval to death, i.e., less than one month after frailty scoring. In this re-analysis, the overall relationship between FI and mortality risk remained strong (HR_age-adjusted_ for mortality per 0.01 unit FI = 1.04 (95% CI = 1.01–1.08; *p* = 0.01)), supporting the robustness of the association between increasing deficit accumulation and mortality risk found in the primary analysis.

## 4. Discussion

Here, we demonstrate that the concept of frailty, operationalized as health deficit accumulation, can be usefully translated to dogs with extreme longevity. EARS-FI, a 34-item clinical FI that captures deficits across multiple domains of health, was developed in dogs of the Exceptional Aging in Rottweilers Study (EARS), a cohort study of dogs reaching extreme longevity. EARS-FI showed several key features observed in aging human populations. First, we documented that deficit accumulation increased with chronological age. Secondly, in age-adjusted risk models, EARS-FI was validated as a predictor of mortality, with greater FI significantly associated with decreased survival. The age-adjusted mortality hazard associated with each 0.01 unit increase in EARS-FI mirrored the relationship between clinical FI and mortality reported in human studies [1,7]. Further support for EARS-FI as a meaningful predictor of mortality risk among dogs with extreme longevity was provided by sensitivity analysis in which dogs with a brief interval to death were excluded from the analysis. Moreover, the frequency distribution of EARS-FI values and the estimated limit of EARS-FI were similar to that reported for clinical FI in human centenarians [5,28]. The research demonstrates that by using readily attainable clinical data collected via standardized telephone interviews with dog owners, we were able to construct an assessment tool that will enable further in-depth analyses of the linkages between deficit accumulation and health outcomes. This investigation of pet dogs in the EARS cohort extends the growing interest in companion dogs as another complementary approach in the growing arsenal of research methods for probing the determinants of healthy longevity.

Frailty index is a marker of biological age that can be used to expose hidden heterogeneity among individuals in an aging population. In dogs experiencing extreme longevity, our results indicate a broad distribution of EARS-FI values, ranging from 0.18 to 0.68, with a median of 0.43. This broad distribution of FI values is consistent with results in human centenarians, with an FI range of 0.00–0.63 (mean, 0.27) in the Chinese Longitudinal Healthy Longevity Survey [28] and an FI range of 0.14–0.73 (mean, 0.47) in an Italian cohort [5]. In contrast, middle-aged dogs [23] and dogs less than 6 years of age [35] had a much lower mean FI, 0.08 and 0.02, respectively. Not unexpectedly, compared to those dogs, our cohort of dogs with extreme longevity had considerably higher FI values with a distribution tending to be right-skewed, as is seen in extreme-aged humans.

An increased vulnerability to death and other adverse health outcomes is associated with an increase in FI. However, there is a limit to the proportion of deficits that can be accumulated by older individuals [33]. It is generally accepted that this limit occurs at an FI value of less than 0.7 [36]. Work by Rockwood and Mitnitski [33] that evaluated the limit of deficit accumulation in more than 33,000 people aged 65+ years living in North America and Australia concluded the 99% limit of FI occurred at 0.65 ± 0.05. This quantifiable submaximal limit to frailty reflects that, in complex systems, when redundancy is exhausted, the system cannot take on additional deficits without failure (i.e., death) [36]. The results presented in the current report, along with previous work in humans [5,28], support the notion that there is a limit to the number of health deficits that can be tolerated by a living organism. This idea of a sustainable FI limit of 0.65–0.7, observed across aging populations, suggests that deficit accumulation may provide a unique window into how organisms cope with cumulative damage.

An important aspect of this study is our validation of EARS-FI as a predictor of mortality risk in dogs with extreme longevity. We showed that increasing values of EARS-FI were associated with a significant increase in mortality risk. For each .01 unit increase in age-adjusted FI, mortality increased by 5%, i.e., HR_age-adjusted_ = 1.05 (95% CI, 1.02–1.08). In a comparative study of the relationship between deficit accumulation and mortality risk in humans and mice, Rockwood and colleagues reported a strong relationship between clinical FI and mortality, with an HR_age-adjusted_ of 1.05 (95% CI, 1.04–1.05) in humans and 1.15 (95% CI, 1.12–1.18) in mice [1]. To express the relationship between a canine FI and 6-month mortality, Banzato et al. reported an HR per 0.01 unit increase in FI of 1.08 (95% CI, 1.06–1.10); it was unclear whether this read-out in dogs was age-adjusted [23]. Taken together, the findings suggest the relationship between FI and mortality in humans may be more similar to that reported in dogs than in mice.

Our data represent the first report on the relationship between deficit accumulation and mortality in dogs with extreme longevity. Reports characterizing frailty in people with extreme longevity, i.e., centenarians, provide an incomplete picture, with several reports lacking a formal description of FI and mortality risk. Interestingly, however, in a cohort of more than 4000 centenarians living in China, the relationship between a 39-item clinical FI and mortality hazard per 0.01 unit increase in FI was 1.016 (95% CI, 1.014–1.018) [29]. These investigators drew attention to their observation that the strength of this association with mortality in human centenarians, although representing a significant impact of FI on mortality risk, was much weaker than in their experience with 65 to 79-year-old study subjects (HR of 1.038 for women, 1.032 for men). Future studies in dogs and humans should attempt to replicate this possible attenuation of the relationship between clinical FI and mortality in individuals achieving extreme longevity. Comparison of the predictive validity of EARS-FI with other frailty tools used in dogs represents one such avenue of inquiry.

EARS-FI was developed using readily attainable information collected non-invasively from dog owners via standardized telephone interviews. Similarly, the canine FI developed by Banzato and colleagues utilized data obtained from owner questionnaire [23]. Taken together, these experiences suggest that a clinical FI obtained non-invasively in dogs can provide a useful tool to interrogate the relationship between frailty and mortality risk. Expanding EARS-FI to include both clinical and laboratory measurement, as has been reported in humans [8] and in mice [37], could provide a platform to gain additional insights into frailty and mortality risk associated with aging.

EARS-FI was developed in a single-breed cohort of dogs that reached extreme longevity. Theoretically, this may limit the translation of this assessment tool’s utility to other breeds or to individuals that experience usual longevity. These caveats are hypotheses that are readily testable by applying EARS-FI, for example, to characterize frailty in exceptionally long-lived individuals of other dog breeds. However, the translatability of this FI to other dog populations was not our primary goal. Instead, our aim was to develop a practical method of capturing clinical data on deficit accumulation that could be put to use directly to support our attempt to uncover clues and generate hypotheses about the regulators of healthy lifespan in a cohort of pet dogs with extreme longevity. We believe we have achieved this aim. The FI reported here has enlarged our investigative toolkit, enriching opportunities for discovery in the EARS longevity study and possibly other aging canine cohorts.

Our study has limitations. Our study of EARS-FI and mortality relies upon cross-sectional data. Therefore, we have not demonstrated the extent to which EARS-FI can be used to study longitudinal changes in frailty. The relatively small size of our analytical sample precluded multivariable analysis from exploring factors that moderate the relationship between deficit accumulation and mortality risk or conducting a detailed inquiry into male-female differences [38] or possible differences associated with gonad removal. EARS-FI was calculated for each dog based on owner-reported deficits. Future studies could be designed to test the extent to which owners may underestimate their dog’s health deficits.

Of utmost importance, our development of EARS-FI offers fresh opportunities for future investigation and potential clinical application. As a readily constructed predictor of mortality risk, EARS-FI may be pivotal in developing new insights into the biological or social factors that can ameliorate (i.e., buffer) the adverse impact of FI on mortality. Currently, this prospect would appear to be within the grasp of investigators, since demographic data from humans support the notion that the adverse impact of any given level of frailty is context-dependent [39]. Also, EARS-FI will enable veterinary researchers to utilize deficit accumulation as an outcome measure in clinical studies. This opens the door to evaluating the impact of lifetime exposures or targeted interventions on overall robustness, as well as the possibility of delaying the advent of clinical frailty.

## 5. Conclusions

Not all dogs of the same breed at the same age have the same vulnerability to the future development of adverse health outcomes. Understanding this biological heterogeneity [40] is one of the keys to advancing the health of geriatric pets. Using data from EARS—the first systematic study of the oldest living dogs in North America—we developed a clinical frailty index, EARS-FI, based upon information readily attained from dog owners through telephone interviews. It was shown that EARS-FI displays key performance features of frailty assessment tools used to characterize aging human populations—a broad range of FI values, an increase in frailty with increasing age, and an upper FI limit < 0.7. Of utmost importance, we demonstrated that increasing deficit accumulation was significantly associated with increased mortality risk in dogs with extreme longevity. EARS-FI will provide a useful tool for further in-depth analyses of linkages between health deficit accumulation and adverse clinical outcomes. This work benefits geriatric pets by contributing to a richer understanding of frailty development in aging dogs and may lead to the identification of factors that undermine optimal trajectories of lifetime physical health.

## Figures and Tables

**Table 1:** List of 34 clinical variables included in the frailty index EARS-FI.

Deficit	Cutoff Values
Decreased appetite	No = 0, Yes = 1
Difficulty chewing or swallowing food or water	No = 0, Yes = 1
Ever able to walk without assistance	Yes = 0, No = 1
Amount of strength detected upon leash walking	* Same = 0, Less = 1
Diminished peak intentional burst of strength	No = 0, Yes = 1
Duration of time spent exercising or playing before tired	Same = 0, Less = 1
Insufficient energy to perform daily physical activity	No = 0, Yes = 1
Diminished eyesight	No = 0, Equivocal deficit causing no problem = 0.5, Yes = 1
Blind in both eyes	No = 0, Yes = 1
Diminished hearing	No = 0, Equivocal deficit difficult to distinguish from selective hearing = 0.5, Yes = 1
Deaf or can hear very loud noises only	No = 0, Yes = 1
Increased frequency of recurrent or symptomatic infection	No = 0, Yes = 1
Inability to control urination	No = 0, Controlled with medication = 0.5, Yes = 1
Inability to control defecation	No = 0, Yes = 1
Amount of time spent sleeping	Same or less = 0, More = 1
Frequency of losing balance	Same = 0, More = 1
Ability to jump	Same = 0, Less = 1
Ability to run	Same = 0, Less = 1
Decreased frequency of engaging in physical activity	No = 0, Yes = 1
Decreased estimated maximum walking speed	No = 0, Yes = 1
Regularly requires assistance to stand up	No = 0, Yes = 1
Exhibits increased aggressive or destructive behavior	No = 0, Yes = 1
Interest in engaging in play	Same = 0, Less = 1
Frequency of confusion, disorientation, or stereotypic behavior	Same or less = 0, More = 1
Able to recognize familiar people	Yes = 0, No = 1
Frequency of appearing painful during or after regular, non-strenuous activities	Same = 0, More = 1
Receives non-intermittent pain medication	No = 0, Yes = 1
Estimated muscle mass (thigh, shoulder)	Same = 0, Less = 1
Poor quality haircoat	No = 0, Yes = 1
Systemic health has declined in the last 3 months	No = 0, Yes = 1
Current Disease Conditions	
Diabetes mellitus	No = 0, Yes = 1
Other endocrine diseases	No = 0, Yes = 1
Cancer	No = 0, Yes = 1
Congestive heart failure	No = 0, Yes = 1
* Same or less represents comparison with function as young adult (3–5 years old).	

**Table 2:** Increase in frailty index (FI) is associated with increased risk of mortality among dogs in the Exceptional Aging in Rottweilers Study (EARS).

	Overall (n = 93)	Females (n = 59)	Males (n = 34)
Age-adjusted HR for mortality per 0.01 unit increase in FI (95% CI)	1.05 (1.02–1.08) *p* = 0.001	1.04 (1.00–1.08) *p* = 0.047	1.07 (1.02–1.13) *p* = 0.004
Median (IQR) FI	0.43 (0.38–0.50)	0.44 (0.38–0.50)	0.41 (0.37–0.51)
Median (IQR) age at frailty scoring	13.2 (13.1–13.6)	13.2 (13.1–13.8)	13.2 (13.1–13.6)

Risk of mortality is presented as an age-adjusted hazard ratio (HR) of mortality associated with a 0.01 unit increase in EARS-FI, a clinical frailty index (see text). Frailty index (FI) and age at frailty scoring are presented as median value and interquartile range (IQR). There were no significant differences in median FI (*p* = 0.89) or median age at frailty scoring (*p* = 0.42) between females and males. 95% CI = 95% confidence interval.

## Data Availability

All data supporting the findings of this study are available within the article and in the Supplementary Table S1. Further inquiries regarding the data can be directed to the corresponding author.

## Supplementary Materials

The following supporting information can be downloaded at https://www.mdpi.com/article/10.3390/ani14243651/s1; Table S1: Table reporting data on sex, age at frailty scoring, frailty index (FI) values, and interval from frailty scoring to death for 93 dogs with extreme longevity in the Exceptional Aging in Rottweilers Study (EARS).

## Abbreviations

EARS, Exceptional Aging in Rottweilers Study; FI, frailty index; EARS-FI, a clinical frailty index developed in the Exceptional Aging in Rottweilers Study
