# Supplementary material for: Frailty and Mortality Risk Among Dogs with Extreme Longevity: Development and Predictive Validity of a Clinical Frailty Index in the Exceptional Aging in Rottweilers Study
Source: Animals (Basel). 2024 Dec 18;14(24):3651. doi: 10.3390/ani14243651 (PMC11672423; doi:10.3390/ani14243651)
Supplement: Supplementary file 1 [file animals-14-03651-s001.zip › animals-3300909-supplementary.pdf]

Supplementary Material

Frailty and mortality risk among dogs with extreme longevity: Development and predictive validity of a clinical frailty index in the Exceptional Aging in Rottweilers Study

David J. Waters, Aimee H. Maras, Rong Fu, Andres E. Carrillo,

Emily C. Chiang, & Cheri L. Suckow

Dog Number	Frailty Index (EARS-FI)	Sex (1-Male; 2-Female)	Age at Frailty Scoring (years)	Interval from Frailty Scoring to Death (months)
1	0.18	2	13.1	6.0
2	0.24	1	13.7	9.9
3	0.26	2	13.2	12.4
4	0.26	2	13.1	13.5
5	0.29	1	13.1	17.5
6	0.32	1	13.6	6.5
7	0.32	2	13.1	23.4
8	0.32	2	13.2	10.4
9	0.32	1	13.0	7.3
10	0.32	2	13.1	9.4
11	0.32	1	13.6	19.8
12	0.34	2	13.1	5.3
13	0.35	2	13.5	13.0
14	0.35	2	13.4	4.0
15	0.35	1	14.0	3.9
16	0.35	2	13.1	11.7
17	0.35	1	13.1	11.4
18	0.35	2	13.0	5.3
19	0.35	1	13.3	5.6
20	0.37	2	13.3	6.2
21	0.38	2	13.3	10.9
22	0.38	1	13.2	10.8
23	0.38	2	13.1	23.1
24	0.38	2	13.8	11.4
25	0.38	2	13.3	17.5
26	0.38	1	13.2	2.9
27	0.38	2	13.2	10.1
28	0.38	1	13.2	3.4
29	0.38	2	13.2	23.4
30	0.38	2	13.0	8.1
31	0.38	1	13.0	12.8
32	0.38	2	13.0	2.8
33	0.40	2	13.7	11.3
34	0.41	2	13.3	0.6
35	0.41	1	13.2	6.9
36	0.41	1	13.0	10.4
37	0.41	1	13.3	23.2
38	0.41	2	13.2	18.6
39	0.41	1	13.6	8.3
40	0.41	1	13.1	4.4
41	0.41	2	13.2	21.1
42	0.41	2	13.1	9.5
43	0.41	2	13.3	24.0

44	0.41	1	13.2	5.8
45	0.41	2	13.8	6.2
46	0.41	2	13.6	5.0
47	0.43	2	13.3	1.7
48	0.44	2	13.8	2.8
49	0.44	2	14.1	16.1
50	0.44	2	13.9	11.1
51	0.44	1	13.3	9.8
52	0.44	1	13.3	4.1
53	0.44	2	13.2	13.5
54	0.44	2	13.1	13.3
55	0.46	2	13.4	20.2
56	0.46	2	13.2	7.6
57	0.46	2	13.0	13.2
58	0.46	1	13.2	8.3
59	0.47	2	14.1	7.5
60	0.47	2	13.8	8.4
61	0.47	1	13.0	9.7
62	0.47	1	13.2	3.6
63	0.47	2	13.1	12.9
64	0.47	2	13.3	4.7
65	0.47	1	13.2	3.8
66	0.47	1	13.6	8.4
67	0.47	2	13.0	1.8
68	0.49	2	13.2	27.4
69	0.50	2	13.3	6.9
70	0.50	1	14.3	9.0
71	0.50	2	13.2	16.6
72	0.50	2	13.2	6.9
73	0.53	2	14.7	1.3
74	0.53	1	13.1	7.2
75	0.53	2	13.1	8.8
76	0.53	2	13.9	6.7
77	0.53	1	13.2	7.2
78	0.54	2	13.0	15.5
79	0.54	2	13.2	1.9
80	0.56	1	14.5	2.5
81	0.56	2	14.6	2.8
82	0.56	1	13.2	7.6
83	0.56	1	13.1	2.5
84	0.56	1	13.1	6.7
85	0.56	2	13.9	0.2
86	0.59	1	13.2	1.8
87	0.62	2	14.1	3.5
88	0.62	2	15.2	1.4
89	0.62	2	13.0	2.1

90	0.65	1	14.8	0.8
91	0.65	2	13.5	0.9
92	0.68	2	14.2	2.1
93	0.68	2	13.8	1.2

Supplementary Table S1. Table reporting data on sex, age at frailty scoring, frailty index (FI) values, and interval from frailty scoring to death for 93 dogs with extreme longevity in the Exceptional Aging in Rottweilers Study (EARS).
